# Acute Presentation and Long-Term Rehabilitation Follow-Up of Ischemic Myelopathy Due to Clinically Suspected Fibrocartilaginous Embolism in an Adolescent Male: A Case Report and Review

**DOI:** 10.3390/neurolint15040080

**Published:** 2023-10-19

**Authors:** Einat Berla, Oleg Kerzhner, Tomm Caspi, Sharon Shaklai, Dianne Michaeli

**Affiliations:** 1 Israel Defense Forces Medical Corps, Ramat Gan 02149, Israel; 2 Loewenstein Rehabilitation Medical Center, Ra’anana 43100, Israel; 3 Sackler Faculty of Medicine, Tel Aviv University, Tel Aviv 39040, Israel; 4 Pediatric Rehabilitation Unit, Department of Physical Medicine and Rehabilitation, Loewenstein Rehabilitation Medical Center, Ra’anana 43100, Israel

**Keywords:** case report, acute myelopathy, fibrocartilaginous embolism, ischemic myelitis, spinal cord infarction

## Abstract

Ischemic myelopathy is uncommon in the pediatric population, with fibrocartilaginous embolism (FCE) being one of its rarest causes. We present the case of an otherwise healthy 17-year-old student who experienced sudden onset of severe low-back pain amidst intensive physical training, which rapidly deteriorated to complete sensory-motor paralysis of his lower limbs. He was treated with IV Methylprednisolone and anticoagulation after the initial work-up suggested spinal cord infarction. After eight days, sufficient clinical-radiological correlation was achieved to support FCE diagnosis as the most likely cause of infarction. He subsequently received inpatient rehabilitation treatment for four months, after which he was followed as an outpatient for a total period of 16 months. While significant neurological and functional gains were achieved during this period, he also experienced some worsening. This case highlights the importance both of performing a thorough assessment and being familiar with FCE as a possible differential diagnosis of spinal cord infarction in children, to facilitate its timely identification and proper acute and long-term management. This case report was prepared following CARE guidelines after obtaining the patient’s written informed consent.

## 1. Introduction

Fibrocartilaginous embolism (FCE) is a rare yet well-established potential cause of non-traumatic spinal cord injury (NTSCI), sometimes termed fibrocartilaginous embolic myelopathy (FCEM) [1,2], and is associated with considerable risk of morbidity and mortality [3]. The first ever description of a spinal cord infarction due to confirmed FCE was provided by a published case report in 1961 [4]. More than 40 additional histologically confirmed FCE cases have been described to date since the seminal report, all of which, except for one, arrived from fatal cases [2]. While histopathological confirmation of spinal cord tissue biopsy remains the only available gold standard for diagnosing FCE, it is highly discouraged in living individuals [5]. Nonetheless, a roughly equal number of spinal cord infarction cases due to clinically suspected FCE, all coming from non-demised individuals, have been reported since 1995 to date, which were mostly made possible only after magnetic resonance imaging (MRI) was introduced into clinical practice [6,7].

The underlying pathomechanisms that account for FCE are not well understood, though it is believed to be caused by dislodged particles of the nucleus pulposus, which embolize and occlude the spinal cord vasculature [2]. Typically, albeit inconsistently, a relatively benign trigger in the form of minor trauma or physical effort is identified in close temporal relation with the sudden onset of spinal cord dysfunction symptoms, which rapidly progress to their nadir [7]. Slight female predominance and a bimodal distribution, peaking in adolescence and late middle age, have also been associated with FCE cases [8].

Due to its rarity, FCE ischemic myelopathy is currently not well-recognized nor well-described, especially for the young [9,10]. It is also believed that it may be a significantly underdiagnosed condition, often confused with more common entities with similar presentation, especially transverse myelitis [3,11]. A better understanding of the distinctive demographic features, clinical course, diagnostic findings, and long-term outcomes associated with FCE myelopathy is necessary to facilitate a better diagnosis and overall management for these patients.

**Figure 1:**
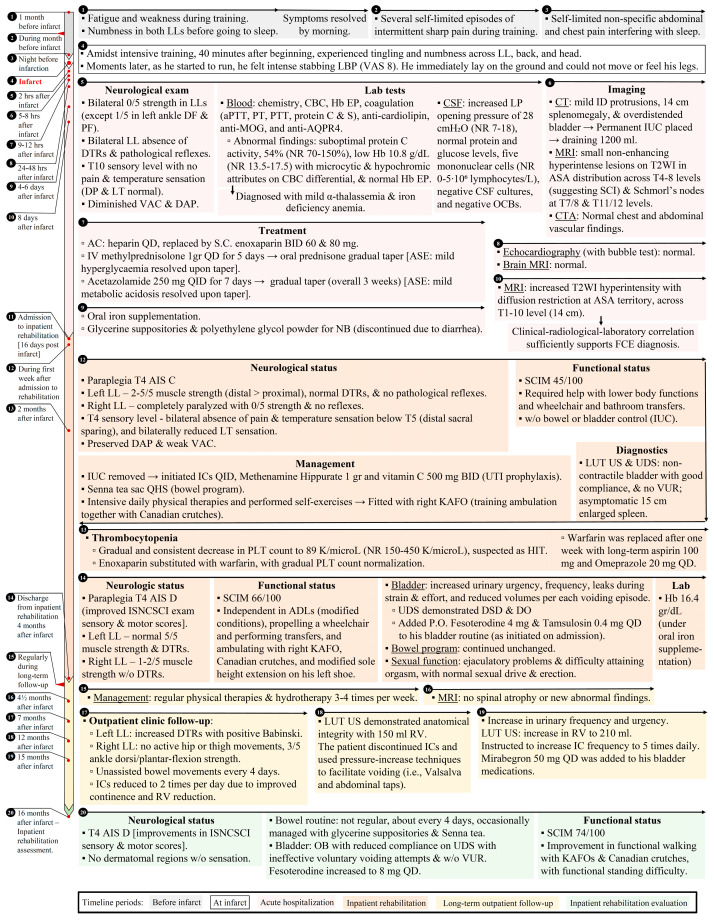
Timeline of key symptoms, clinical features, interventions, and outcomes from the initial identification of associated symptoms one month prior to infarction until 16 months after. **Abbreviations:** AC, anticoagulation; ADL, activities of daily living; anti, antibodies; aPTT, activated partial thromboplastin time; ASA, anterior spinal artery; ASE, adverse side effect; AQPR4, aquaporin 4; BID, “bis in die” (Latin, twice a day); CBC, complete blood count; CSF, cerebral spinal fluid; CT, computed tomography; CTA, computed tomography angiography; DAP, deep anal pressure; DF, dorsiflexion; DO, detrusor overactivity; DP, deep pressure; DTR, deep tendon reflexes; DSD, detrusor sphincter dyssynergia; DWI, diffusion-weighted image; EP, electrophoresis; Hb, hemoglobin; HIT, heparin-induced thrombocytopenia; IC, intermittent catheterization; ID, intervertebral disc; ISNCSCI, International Standards for Neurological Classification of Spinal Cord Injury; IV, intravenous; KAFO, knee-ankle-foot-orthosis; Lab, laboratory; LBP, low back pain; LL, lower limbs; LP, lumbar puncture; LT, light touch; LUT, lower urinary tract; MOG, myelin-oligodendrocyte glycoprotein; MRI, magnetic resonance imaging; NB, neurogenic bowel; NR, normal range; PF, plantar flexion; PLT, platelets; PT, prothrombin; PTT, partial thromboplastin time; QD, “quaque die” (Latin, once a day); qhs, “quaque hora somni” (Latin, every night at bedtime); QID, “quater in die” (Latin, four times a day); RV, residual volume; TID, “ter in die” (Latin, three times a day); OCB, oligoclonal bands; S.C. subcutaneous; SC, spinal cord; SCI, spinal cord infarct; VAC, voluntary anal contraction; VUR, vesicoureteral reflux; UDS, urodynamic studies; UTI, urinary tract infections.

Herein, we describe the case of an otherwise healthy 17-year-old high school student admitted to inpatient rehabilitation after being diagnosed with a spinal cord infarct due to clinically suspected FCE. After discharge, the patient continued to be treated in outpatient and community settings and was followed for a total period of 16 months (Figure 1, timeline summary). This case report was prepared following the CARE guidelines (Supplementary Figure S1) after obtaining the patient’s consent [12].

## 2. Case Presentation

This healthy 17-year-old Caucasian male of Jewish descent was a physically active high school student who regularly participated three to four times a week in an intensive extracurricular training program preparing for military service. One month before acute admission, he experienced non-specific and transient fatigue and general weakness during a training session. Later that day, he noticed weakness and numbness in both his lower limbs (LL) upon lying in bed before falling asleep, which resolved by the morning. Intermittent and non-consistent sharp pain in his back and LLs was also experienced during several training sessions. The night before his admission, he experienced a self-limited short episode of non-specific abdominal and chest pain that interfered with his sleep. On the next day, he participated in a training session that included alternating sets of short-burst aerobic and power exercises (i.e., running, push-ups, sit-ups, and supine leg paddles). After 40 min, when he was about to perform another set of short runs, he began to experience a tingling and numbness sensation across his LL, back, and head. As he began to run, he immediately felt a sudden onset of intense stabbing sharp pain, localized to his lower back, which he describes as the most severe pain he has ever experienced, grading it as an eight on the visual analog scale (VAS). He immediately lay on the ground and noticed a few seconds later that he was unable to move or feel his legs. Subsequently, he was immediately rushed to a nearby emergency room. Except for an uncomplicated appendectomy performed at the age of 8 years, his medical and family history was unremarkable, and he did not experience any recent serious trauma, fever, infectious episodes, or initiating new drugs or therapies.

### 2.1. Acute Hospitalization

Upon admission, the patient was hemodynamically stable, and his vital signs were within normal range. Neurological exam findings did not reveal mental status or cranial nerve deficits. A motor exam demonstrated bilateral symmetric flaccid paralysis with 0/5 strength across his LLs, with the exception of residual motor function preservation noticed at the left foot with measured strength of 1/5. Pathological and deep tendon reflexes (DTRs) were absent bilaterally. A sensory level with absence of pain and temperature sensation beginning at the T10 dermatome was noticed, with preserved proprioception, deep pressure (DP), and light touch (LT) sensations. The rectal tone was significantly diminished, as was the anal sensation. The patient was no longer in pain and was neurologically stable.

**Figure 2:**
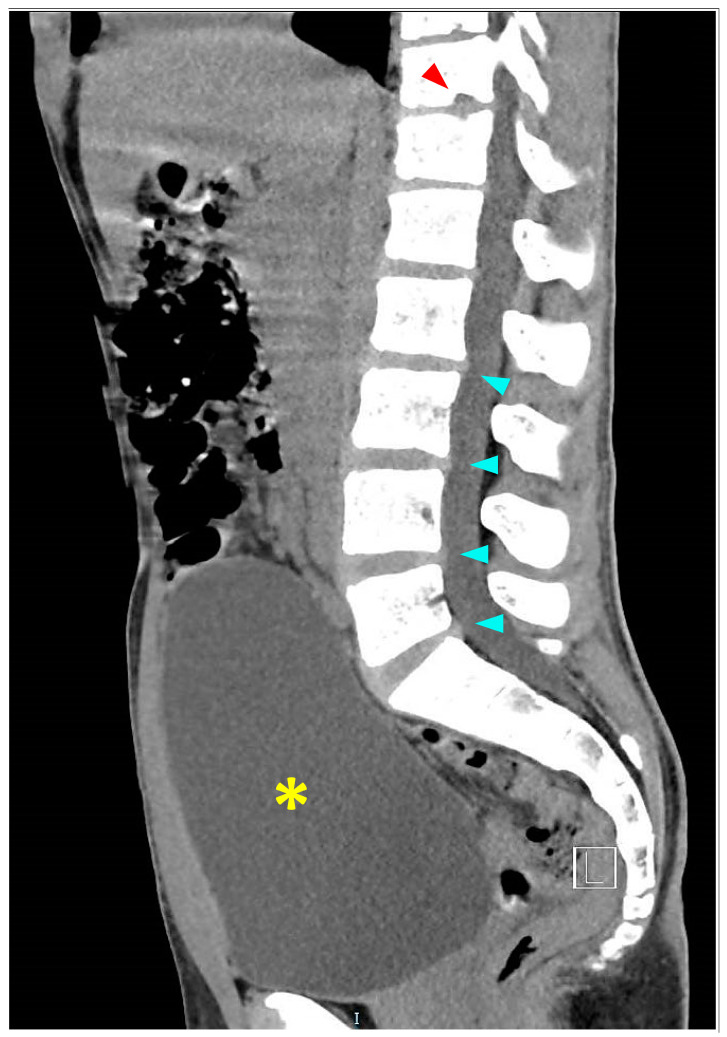
Sagittal view of thoracolumbar CT scan performed five hours after infarct demonstrating mild intervertebral disc protrusions (turquoise arrowheads), significantly overdistended bladder (yellow asterisk), and Schmorl’s node (red arrowhead).

**Figure 3:**
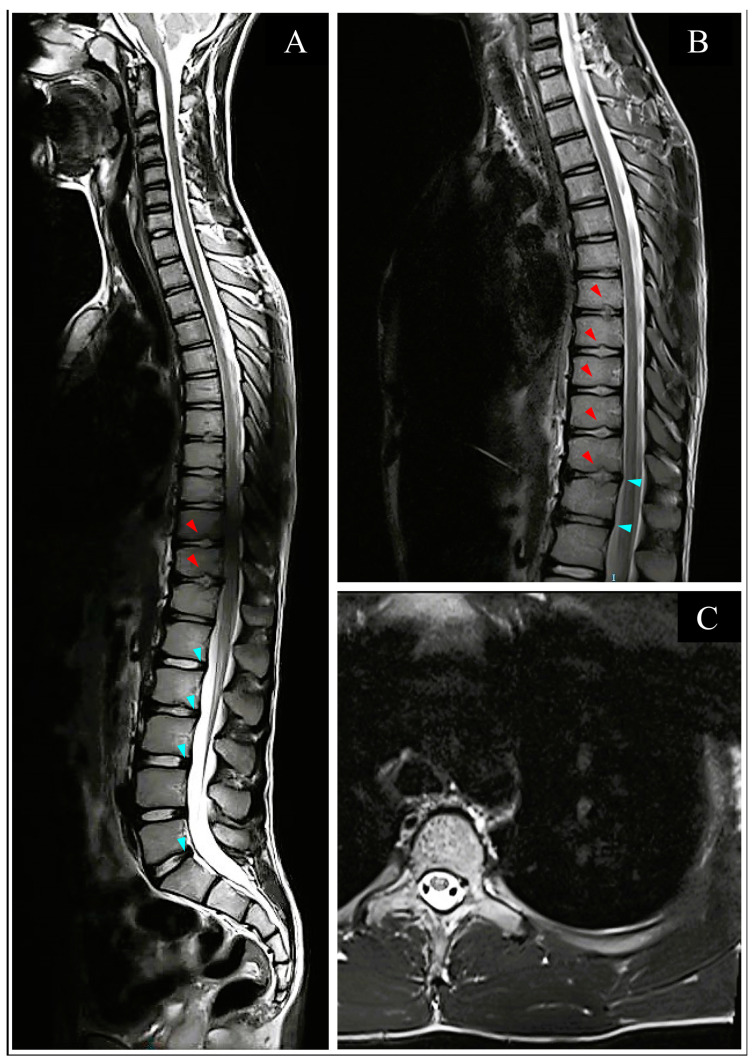
T2-weighted MRI performed 7 h after infarct. (**A**). Sagittal T2-weighted MRI of cervical-thoracic-lumbar spine performed 7 h after infarct. Note the mild horizontal disc protrusions (turquoise arrowheads) and Schmorl’s nodes (red arrowheads). (**B**). Close-up image of T2-weighted thoracolumbar spine also demonstrates mild horizontal disc protrusions (turquoise arrowheads) and Schmorl’s nodes (red arrowheads). (**C**). Axial view of the thoracic spine, about T5 level, demonstrating two small hyperintense lesions at the anterior part of the spinal cord, “owl’s eyes” pattern, suggesting infarction.

An initial thoracic and lumbar spinal cord computed tomography (CT) scan demonstrated mild disk protrusions producing minimal pressure against the dural sac at several lumbar levels with no evidence of spinal stenosis and other intervertebral disc (ID) abnormalities (Figure 2). A significantly overdistended bladder was demonstrated on CT (Figure 2), with residual urine of 1200 mL measured and released by an indwelling urinary catheter (IUC) placed after the scan and left throughout the rest of the hospitalization. Splenomegaly of 14 cm was also noticed and confirmed on a subsequent abdominal ultrasound (US). Subsequent thoracolumbar spine magnetic resonance imaging (MRI) demonstrated small non-enhancing hyperintense lesions on T2 weighted image (T2WI) in the anterior aspect of the cord between the T4 and T8 levels, suggesting anterior spinal artery (ASA) distribution cord infarction (Figure 3C). Schmorl’s nodes were identified at the T7/8 and T11/12 levels by the typical pattern of vertical herniation of intervertebral disc cartilage material into the adjacent bony upper and lower vertebral bodies (Figure 3A,B). Significant horizontal ID herniations were not noticed. Normal chest and abdominal CT angiography (CTA) findings excluded possible vascular causes (i.e., aortic dissection).

Serum chemistry, complete blood count (CBC), coagulation profile (activated partial thromboplastin time, protein C and S levels, and prothrombin), and autoimmune studies [anti-cardiolipin antibodies, myelin-oligodendrocyte glycoprotein antibody (anti-MOG), and anti-aquaporin 4 antibody (anti-AQPR4)] were performed. Noteworthy findings included suboptimal protein C activity at 54% [normal range (NR) 70–150%] and low hemoglobin of 10.8 g/dL (NR 13.5–17.5 g/dL) with microcytic and hypochromic attributes on the CBC differential. With non-remarkable hemoglobin electrophoresis results, alpha thalassemia combined with iron deficiency anemia were diagnosed as part of hematologic consult and follow-up. Spinal lumbar puncture (LP) demonstrated mild increased opening pressure of 28 cmH_2_O (NR 7–18 cmH_2_O), and unremarkable cerebral spinal fluid (CSF) analysis with normal protein and glucose levels and five mononuclear cells (NR 0–5∙10^6^ lymphocytes/L). CSF microbiology cultures were negative, and oligoclonal bands (OCBs) were not identified.

Neurosurgical consultations from two hospitals advised against attempting ASA revascularization due to lack of proven efficacy. Anticoagulation treatment with heparin was initiated and replaced with subcutaneous (S.C.) enoxaparin twice daily (60 and 80 mg). High-dose intravenous (IV) pulse regimen of methylprednisolone 1 gr/day was acutely initiated for five days. Without any noticeable clinical improvement, it was gradually tapered by oral prednisone. Acetazolamide 250 mg 4 times per day was also acutely initiated and later was gradually tapered, completing a 3-week treatment. Oral iron supplementation was also initiated. Neurogenic bowel was managed with glycerin suppositories and polyethylene glycol powder, which were discontinued prior to discharge due to diarrhea.

**Figure 4:**
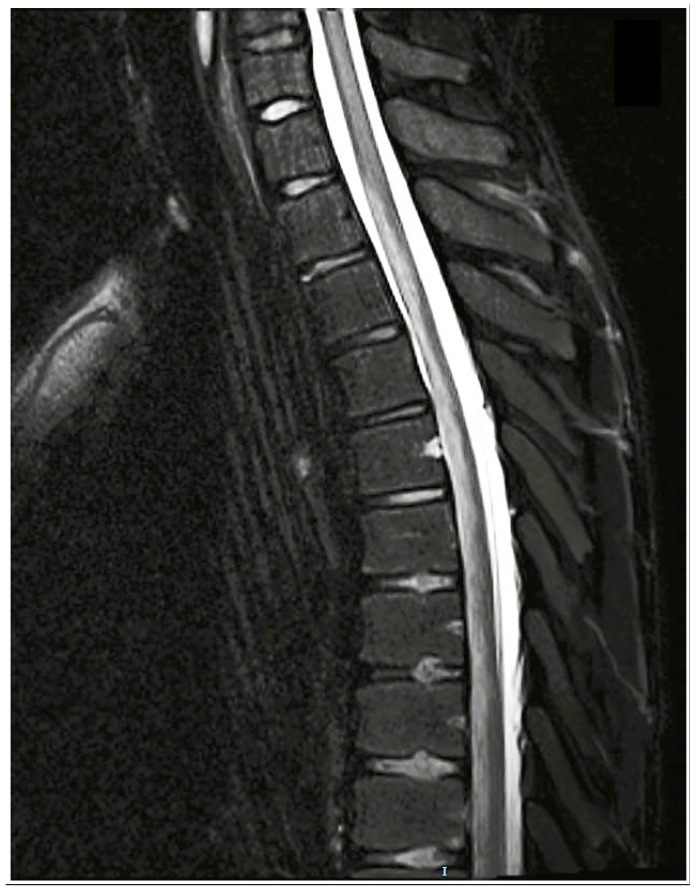
Sagittal T2-weighted MRI of cervical-thoracic spine performed 8 days after infarct demonstrating increased hyperintensity at ASA territory, from T1/2 to T10 level, spanning 14 cm in length, and was not demonstrated at admission (Figure 2).

Further thromboembolic workup was unremarkable by cardiac echocardiography with a bubble test and brain MRI. Eight days after admission, a repeat MRI demonstrated increased T2WI signal hyperintensity with diffusion restriction on the diffusion-weighted image (DWI) at ASA territory, extending from T1/2 to T10 level about 14 cm in length (Figure 4). These findings and the overall clinical–laboratory–radiological correlation led to the assumption that the spinal cord infarction could have most likely resulted from FCE. Repeat blood tests were noticeable for metabolic acidosis and mild hyperglycemia, most likely secondary to acetazolamide and steroidal treatment, respectively, as both resolved upon tapering of these medications.

During the remaining period of the acute stay, there was some progress in the patient’s ability to move his left foot and knee, and a positive Babinski sign on that side, with no improvement demonstrated on the right side. The sensory level has moved from T10 to T7 level with a complete absence of pain and temperature sensation.

### 2.2. Inpatient Rehabilitation

Sixteen days after the acute infarct, the patient was admitted to a rehabilitation center for inpatient rehabilitation treatment and care at a specialized spinal cord rehabilitation unit. A motor exam demonstrated incomplete left LL paralysis, with muscle power grade of 2/5 to 5/5, with proximal being weaker than distal muscle groups, normal DTRs, and no pathological reflexes. The right LL was completely paralyzed with 0/5 strength and no reflexes. T4 sensory level with bilateral absence of pain and temperature sensation below T5, with sparing of the most distal sacral segments, was observed. LT sensation was bilaterally and symmetrically reduced below the T5 level. Preserved deep anal pressure (DAP) sensation and weak voluntary anal contraction (VAC) were demonstrated. The patient was subsequently classified as paraplegia T4 AIS C. There was no control over bowel functions, and a permanent IUC was used for managing his bladder dysfunction. While upper body self-care was independently managed, lower body care, as well as performing wheelchair and bathroom transfers, required assistance. At admission, a spinal cord independence measure (SCIM) score of 45/100 was given.

Lower urinary tract (LUT) US and urodynamic studies (UDS) demonstrated noncontractile bladder with good compliance, absence of vesicoureteral reflux (VUR), and complete inability to contract upon voluntary voiding attempts (Table 1). An enlarged spleen of 15 cm was also demonstrated. The permanent urinary catheter was removed, and the patient initiated an intermittent self-catheterization (IC) routine, performed 4 times a day. He gradually regained some urinary voluntary control and continence with a parallel decrease in IC frequency to 3 times per day. Methenamine Hippurate 1 gr and vitamin C supplement tab 500 mg were given for urinary tract infections (UTIs) prophylaxis twice a day. The bowel program was managed with one Senna tea sac per day.

Two months after the infarct, a gradual and consistent decrease in platelet (PLT) count was noticed, reaching 89 K/microL (NR 150–450 K/microL), suspected as heparin-induced thrombocytopenia (HIT). After a hematological consultation, the patient was transferred to warfarin, and enoxaparin was discontinued, which halted the drop in PLT counts and resulted in their gradual normalization. Warfarin was discontinued after a week and replaced with long-term aspirin 100 mg once a day, after normal results on repeat LAC and JAK2 assays, together with Omeprazole 20 mg per day for gastroprotection.

The patient demonstrated high motivation, participated regularly in intensive daily physical therapy (PT) treatments, and performed self-exercises during the day. A right knee-ankle-foot-orthosis (KAFO) was fitted, and continuous functional and neurological improvements were demonstrated during his inpatient stay. The patient developed a slight increase in his left lower limb muscle tone, without functional disturbances, while towards the end of hospitalization, experienced an increase in the flexor tone of his right foot second toe, which caused disturbance upon rest and ambulation and was managed with a Botox neuromuscular block attempt.

Four months after the insult, due to complaints of increased urinary urgency, frequency, leaks during strain and effort, and reduced volumes per each voiding episode, a repeat UDS was performed one week prior to discharge with findings of detrusor sphincter dyssynergia (DSD) and overactivity (DO) (Table 1). Once a day oral Fesoterodine 4 mg and Tamsulosin 0.4 mg treatment was initiated. The patient was instructed to maintain at least three ICs a day and to perform an additional one in case urinary volume exceeds 400 mL while avoiding as much as possible applying exaggerated intra-abdominal pressure during voiding attempts. Further assessments and follow-ups in the sexual rehabilitation clinic were performed due to ejaculatory problems and difficulty reaching orgasm, while his sexual drive and erection were intact.

The patient completed 107 days of inpatient rehabilitation. During this period, normal strength in his left LL was regained, with a more modest recovery in the right LL, with 1–2/5 muscle strength and no DTRs. His sensory level remained at the T4 level, though both sensory and motor scores on the International Standards for Neurological Classification of Spinal Cord Injury (ISNCSCI) exam improved, and he was classified as a T4 AIS D upon discharge. SCIM of 66/100 was scored at discharge as functional gains were also achieved. The patient achieved independence in his activities of daily living (ADLs) under modified conditions, was independently propelling a wheelchair and performing transfers, and was able to walk with right KAFO, Canadian crutches, and modified sole height extension on his left shoe. His bowel routine was maintained with the help of one Senna tea bag a day and 3 times a day ICs, together with continuing Fesoterodine and Tamsulosin treatment for DSD management and vitamin C and Methenamine Hippurate. His hemoglobin levels normalized to 16.4 gr/dL, and long-term oral treatment with Folex 400 mg per day after discharge was prescribed. The patient was discharged with a long-term outpatient treatment and follow-up program, which included various medical specialists, and was scheduled to return for a routine overnight inpatient medical and functional status assessment in our medical rehabilitation center.

### 2.3. Long-Term Follow-Up

**Figure 5:**
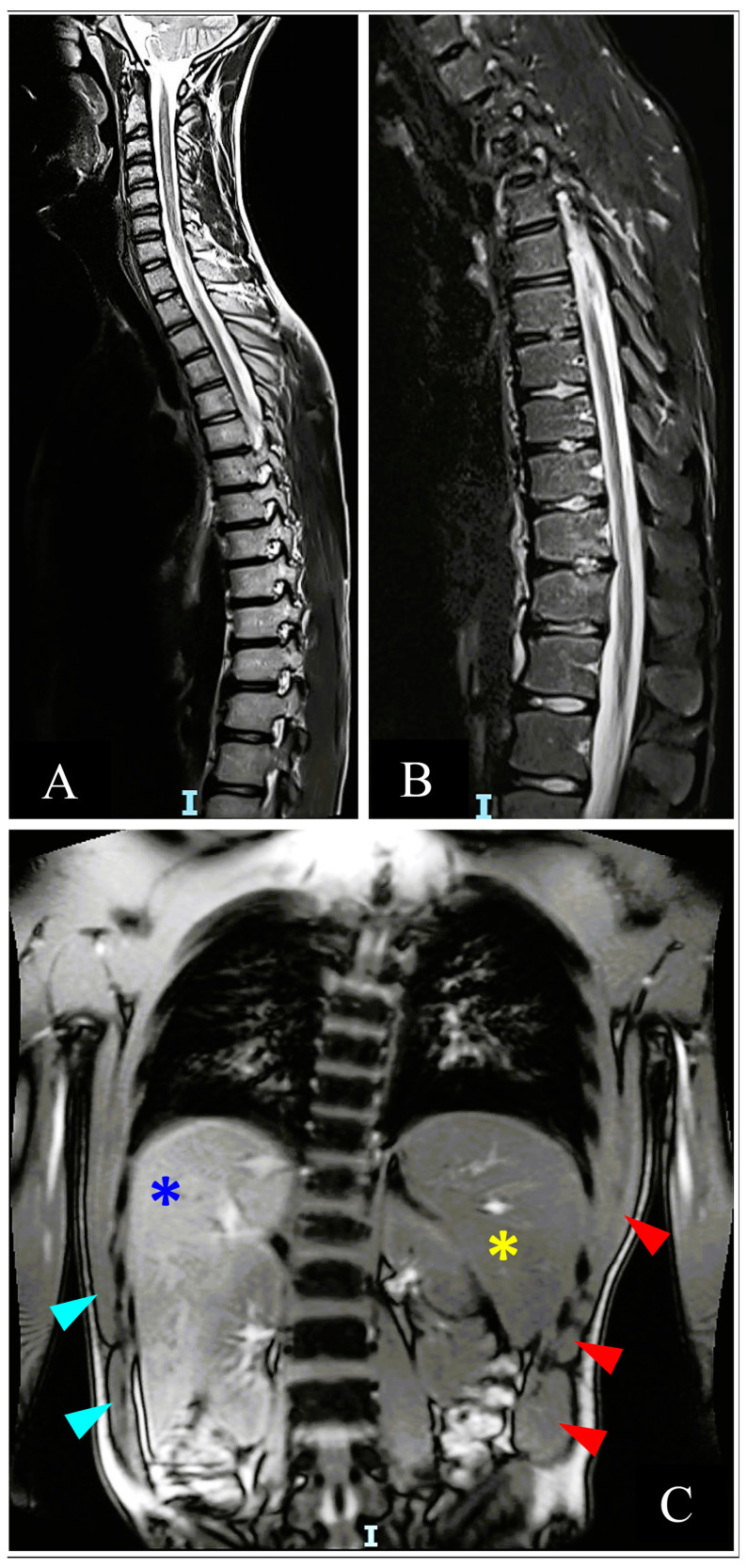
T2-weighted MRI performed four and half months after the infarct. (**A**). Sagittal view of cervical-thoracic cord not demonstrating spinal atrophy or new abnormal findings development during interval. (**B**). Sagittal view of more caudal parts of the cord compared to 2A, demonstrating similar findings. (**C**). Coronal view demonstrates splenomegaly (yellow asterisk) and mild hepatomegaly with elongated liver span (blue asterisk), which align with the patient’s new alpha-thalassemia diagnosis. Relative atrophy of lateral abdominal and chest wall muscles on the left side (turquoise arrowheads) compared to the right side (red arrowheads), which are in line with the patient’s significant lower limb weakness on the right side. Scoliosis is also demonstrated.

Thoracolumbar MRI four and a half months after the insult showed no evidence of spinal atrophy or new abnormal findings (Figure 5). He performed regular PT and hydrotherapy treatments three to four times a week, showing steady progress toward established goals, and was able to slightly move his right foot. The flexor tone in his right foot’s second toe continued to increase, leading to discomfort and pain. Concurrently, he began experiencing bilateral LL intermittent cramps, which were more pronounced on the right, and right foot sensory abnormalities such as numbness, paresthesia, and burning sensation.

Three months post-discharge, during neurological and rehabilitation outpatient clinic follow-up, an increase in left lower limb DTRs was noticed with the persistence of the toe extensor reflex response (Babinski). No active hip or thigh movements were observed on the right, while progress to 3/5 ankle dorsi/plantar-flexion strength was observed. Unassisted bowel movements were experienced about every four days. The patient reduced ICs to two times per day as he experienced an improvement in his continence and a reduction in residual volumes. Ultrasound performed eight months post-discharge demonstrated LUT anatomical integrity with 150 mL residual volume.

After several neurologic, rehabilitative, and orthopedic follow-ups, further Botox injection into the second right toe was discouraged as the increase in tone was only observed for an already-weak tibialis anterior at ankle dorsiflexion, thus trying to avoid any loss of its residual strength. The patient discontinued ICs as he felt it was interfering with social participation and used intrabdominal pressure increase techniques such as Valsalva and abdominal taps. Increased frequency and urgency were still experienced, while urinary leaks rarely occurred. Some improvement in attaining orgasm was also noticed. Subsequent LUT follow-up US performed eleven months after discharge demonstrated an increase in residual volume to 210 mL (Table 1). The patient was guided to increase IC frequency to 5 times daily, and Fesoterodine was added with Mirabegron 50 mg daily.

### 2.4. Repeat Inpatient Follow-Up

The patient was admitted to an overnight inpatient rehabilitation assessment one-year post-discharge and 16 months after the infarct. He was classified as T4 AIS D, demonstrating motor and sensory score improvements with no dermatomal regions of anesthesia for anterior spinal sensory modalities. SCIM score of 74/100 reflected the significant improvement in functional walking abilities with the KAFOs and Canadian crutches, which were reported and demonstrated, while functional standing was still difficult. Improving the latter and uneven terrain ambulatory abilities were set as further treatment goals. His blood tests were normal, with no signs of anemia. His bowel routine was not regular, occurring every 4 days, with occasional management with glycerin suppositories and Senna tea. He reported that he recently returned to regularly taking prescribed LUT medications and performing ICs as recommended while still feeling the need to exert a lot of effort during voiding. UDS demonstrated OB with reduced compliance, reaching abnormally high pressures upon voluntary voiding attempts without effectively initiating micturition (Table 1). VUR was not demonstrated, and the Fesoterodine dosage was increased to 8 mg per day. A follow-up visit and VUD study in three months was scheduled for him.

## 3. Discussion

### 3.1. Epidemiology

Of all acute myelopathic cases, 5% to 8% are accounted for by ischemic etiologies [13,14,15], of which 1.5% to 5% were previously reported to be due to FCE [2,8,10,13,16]. As such, acute FCE ischemic myelopathy is a rare condition mostly reported by case reports and small case series [7,8]. For the most part, these demonstrate a bimodal pattern of presentation, with a peak incidence in adolescence and middle age [7,9,17]. However, upon acutely presenting non-traumatic myelopathy, clinically overlapping conditions that are both more common and better recognized are usually suspected first [3,18] Inflammatory and autoimmune conditions, especially transverse myelitis, are the pediatric population’s immediate culprits [7,11]. Moreover, the substantially lower rates of pediatric SCI in general, and those of non-traumatic ischemic nature specifically compared to the adults, further underlie the low clinical suspicion of FCE and undermine its timely and accurate diagnosis and management [3,9,19,20]. These issues have led some authors to assume that FCE is an underdiagnosed condition [2]. For the patient in our case, the identification of FCE as the most likely cause of his spinal infarct was made eight days from acute admission after a sufficient amount of diagnostic work-up ruled out the other possible etiologies. Few reviews have previously assessed the pediatric FCE cases specifically, reporting up to 26 identified cases overall. Contrary to the roughly similar sex distribution for adult cases, 3:2 female predominance was reported, the reason for which is not well understood [3,9,11].

### 3.2. Pathophysiology

Complete understanding of the underlying mechanism and risk factors for FCE are not well understood [7,9]. The held theory for FCE development is believed to result from an initial dislodgement of fibrocartilaginous material from the inter-discal nucleus pulposus [10]. This event requires a sudden increase in axial and intradiscal pressure, possibly due to some physical exertion or minor trauma. When combined with increased internal pressure, such as during Valsalva, the dislodged particle may migrate retrogradely within terminal vertebral arterial feeders and enter more proximal parts of the spinal arterial system [8,21]. Systemic access to the embolic fragment is provided by abnormal vascular supply to the intervertebral cartilage, an otherwise avascular structure under normal conditions [18]. It is postulated to result from either persisting elements of neonatal disc vasculature or neovascularization processes associated with age-related degeneration of spinal column elements [2,7]. Several authors argue in favor of another theory, in which the ID herniates vertically into vertebral body marrow, termed Schmorl’s nodes, which may provide systemic access via its valveless venous sinusoids to a dislodged cartilaginous material during significant intrathoracic pressure rise [11,18,22]. The subsequent migration of the embolic particle into spinal vasculature may result in an overt occlusion at a specific segment and is believed to account for the development of ischemic myelopathic damage [1,18]. Schmorl’s nodes were previously perceived as evidence of age-related degenerative change and were discussed as a possible attributive risk factor for FCE in the adult population, however, it is now recognized that they may well appear also in the young, with up to 24% of reported cases below age 18 had this finding [2,7,9]. When looking at any kind of noticeable structural abnormality of the vertebral column’s bony or cartilaginous components (i.e., horizontal disc herniation), it can be expected to occur in more than two-thirds of the pediatric population [9,23]. As such, these findings have been repeatedly suggested as FCE-supportive diagnostic findings for adults and children [3,7,8,22].

### 3.3. Diagnosis

As FCE definite diagnosis is made by postmortem histological conformation of the tissue sample, an antemortem definite diagnosis of surviving patients is not usually possible [21], since cord biopsy is associated with a high risk of exacerbating the existing neurological damage and it is highly discouraged [24,25]. Nonetheless, several studies in recent decades proposed several diagnostic algorithms for identifying FCE ischemic myelopathy with high probability [7,8,16]. Thus, FCE diagnosis in surviving patients is one made by exclusion [7,18,24]. Using a stepwise approach for ruling out alternative diagnoses, FCE diagnosis could be confidently assumed after achieving a certain beyond-threshold constellation of key clinical and radiological correlation [7,8,16].

#### 3.3.1. Clinical Presentation

The usually described clinical presentation often commences with transient neck or back pain, rapidly succeeded by progressing neurological deficits that reach their nadir within hours of the identified trigger [8,21]. The patient in our case described experiencing tingling and a sensation of numbness across his limbs, back, and head amidst intensive physical training, which included adjacently alternating exercises involving axial strain (jumping and running) and increased intrathoracic pressure (back-pedaling and sit-ups). A few minutes later, as he began to run again, he felt a sudden onset of severe and sharp lower back pain which was followed by complete motor and sensory paralysis of his lower limbs within seconds. This described combination of suddenly appearing severe back or neck pain in close temporal association with an identified trigger, in the form of physical effort or minor trauma performed together with a Valasalva-type maneuver, is considered very characteristic of FCE. However, this presentation is not consistent, nor it is mandatory for diagnosis [7,8]. Identified traumatic triggers in 24% and performing intense exercise in up to 50% of pediatric cases was previously reported ^11^. According to the spinal vascular territory affected, various symptoms may ensue, most commonly being those of anterior spinal artery occlusion [7,11,21]. The latter usually presents with a bilateral motor deficit, though asymmetrical presentation is possible, bladder or bowel dysfunction, and sensory deficit of spinothalamic modalities [7,8,9,13]. The rapid onset and progression of all of these symptoms in our patient are considered highly supportive for ischemic over inflammatory causes and considered a good clinical indicator for FCE etiology [8,9,16].

#### 3.3.2. Imaging

Imaging studies performed upon initial evaluation of acute nontraumatic myelopathy usually include CT and CTA as rapid modalities for evaluating vascular or structural causes, followed by MRI scans to detect possible ischemic or inflammatory findings [11,22,26]. Typical findings in the early stages of clinical onset that strongly support FCE etiology are normal vascular findings on CT/CTA scans, a non-enhancing increased T2 signal, and swelling of the cord on MRI, together with additional structural findings of associated narrowed intervertebral disk or Schmorls’ nodes which may be observed on both modalities [18,24,27]. However, as these acute ischemic changes of FCE may not be visible on MRI in the first 24 to 48 h, differentiating ischemic from inflammatory etiologies, and especially from idiopathic transverse myelitis can be very difficult [11,22,28]. However, when clinical suspicion is high, and T2 findings are negative, DWI has been reported as a good option for early detection, even within several hours from the onset, owing to its high sensitivity for identifying infarcted areas [29]. Unremarkable CT and CTA results in our patient, performed about 5 h after acute onset, were followed by T2WI protocol which demonstrated anterior cord territory small hyperintensities, with both modalities demonstrating Schmorl’s nodes and horizontal herniations. Diffusion restriction on DWI with T2WI hyperintensity extending from T1/2 to T10 level was demonstrated on MRI performed eight days from acute onset to assess final damage. The anatomical distribution of infarcts in pediatric FCE cases did not seem to follow a specific pattern and was reported to occur across all spinal levels [11].

#### 3.3.3. Laboratory Tests

CSF analysis results are also important to facilitate FCE diagnosis as the most likely cause for acute myelopathy [8,18]. Elevated protein levels are expected, though normal results could also be observed [11]. FCE can be differentiated from other prevalent causes of non-traumatic myelopathies by the absence of specific CSF findings such as pleocytosis, oligoclonal bands, or an elevated IgG index, which are characteristic of inflammatory conditions like autoimmune or infectious diseases [8]. Our patient’s initial blood tests demonstrated mild alpha thalassemia combined with iron deficiency anemia and sub-optimal protein C activity with otherwise normal coagulation studies. However, of these factors, only the latter have been previously associated with an increased risk of ischemic myelopathy in general [28]. On the other hand, the increased risk of FCE was not previously associated with any of these conditions [7,9,11].

Moreover, although more common in the severe forms of thalassemia, elevated levels of various circulating vascular growth factors, are known to account for the various vascular trophic effects and their associated clinical manifestations [30]. The most common findings are hematopoietic and lymphoid tissue proliferation, such as hepatomegaly and splenomegaly [31,32]. Our patient also had consistent findings of non-dynamic, and otherwise asymptomatic, splenomegaly repeatedly demonstrated across all US tests. It is not unreasonable to assume that if intervertebral disc vasculature abnormally persisted into adolescence in our patient, it may have been facilitated to a certain extent by a similarly shared angio-proliferative mechanism with a possible additive effect by the iron deficiency anemia, which he was also diagnosed with. We were able to find only one reported case, in all age groups, of FCE with concomitant hemoglobinopathy, which occurred in a 12-year-old girl with hemoglobin SC disease [23]. While deriving any casual or associated risk-attributive properties to this relationship is currently impractical due to the extreme paucity of reported cases [33], it represents a potentially important knowledge gap for future studies [7,11]. Alongside genetic disorders, acquired coagulation disorders are also known for predisposing to spinal cord infarctions. Of these, dehydration is especially intriguing, as many FCE cases, including in our patient, occur during physical effort, which also raises some questions about possible associations for future studies to examine [34].

### 3.4. Treatment and Prognosis

#### 3.4.1. Acute Phase Period

Currently, no specific treatment is available for spinal cord infarcts in general and FCE specifically, which are managed by various non-specific measures in acute and long-term settings [11,34]. During the initial acute period, when diagnostic work-up did not exclude vascular or inflammatory causes, treatments such as corticosteroids and other immunosuppressive measures, anticoagulants, and surgical interventions have been consistently reported to be used [7]. The former also may ameliorate the acute spinal edema associated with spinal ischemia [8,16,28] and was given to our patient together with Acetazolamide for that purpose. Our patient was also treated with a short course of anticoagulation and then was transferred to several months of oral anti-platelet agent. The latter can be recommended in cases suspicious of involving small vessel thrombosis, however, similarly to the steroids, there is no proof that these treatments are of any benefit [28,34]. Nonetheless, these treatments are still routinely provided as a standard of care until other etiologies have been excluded, as they are relatively safe when given for a short period, and the associated risk is outweighed by the potential harm of non-treating [7,8]. Higher levels and extensiveness of spinal infarct seem to be the only consistent factors to be cited as associated with worse outcomes in the acute phase, specifically in FCE [7,11]. While this is in line with the current knowledge concerning recovery from ischemic myelopathies, under which FCE is classified [11,27] it is also important to recognize that these observations do not rely on evidence-based studies, but rather mostly on individually reported cases [9,34].

#### 3.4.2. Long-Term Period

Long-term interventions and outcomes are also not well described, and for the most part, are mentioned very generally and briefly [7,8,28,34]. The importance of rehabilitative medical and physical treatments in preventing medical complications and improving functional and quality-of-life outcomes was previously recognized [3,7,11,27]. These interventions, if described, are usually mentioned for being non-specific and provided with limited descriptions such as in- or out-patient rehabilitation, physiotherapy, or just mentioning admission to rehabilitation. Lack of evidence to support their efficacy is also repeatedly cited [2,7,8]. Higher levels and extent of spinal cord infarction are similarly associated with worse long-term outcomes [11,28]. However, these are described mostly by imaging findings and gross motor and sensory neurological exam findings by grading for example 0 to 5 on the Medical Research Council scale (MRC) for muscle strength grading or describing the sensory modality as absent or impaired, respectively. We found 18 studies describing pediatric FCE cases admitted to rehabilitation, out of which only three were at least once provided with classification according to the ISNCSCI [35,36,37], which is the international gold standard (GS) for SCI neurological status evaluation [38]. The latter facilitates in quantitative assessment of SCI degree (AIS grade) by systematically evaluating sensory and motor deficits, which together with the anatomical level of injury can be used to predict neurological and functional outcomes, follow long-term progress, assess treatment efficacy, and guide further interventions and treatment goals [39,40].

Our patient was followed for 16 months from the acute event and was a T4 AIS C upon admission to rehabilitation (Figure 1). We observed continuous long-term improvement in sensory and motor scores, while the neurological level did not improve beyond the first initial weeks. The patient was T4 AIS D at discharge from inpatient rehabilitation as well as upon his last follow-up, with both motor and sensory scores showing improvement (Table 1). Understanding of the benefits of the various interventions we described compared to other cases, as well as the neurological improvement, is limited due to the abovementioned issues of non-standardized reporting. Our patient demonstrated limited improvement in sexual and bowel function while at the same time experiencing deterioration in bladder function. The patient had OB with normal compliance with good continence, which was managed by oral medications and daily IC routine. After a year, reduced compliance to bladder management care due to self-perceived improvement led to deterioration with increased residual volumes and lower compliance of the bladder. His bowel function and routine were also not satisfactory at the long-term follow-up. We did not find long-term follow-up being reported for the latter or for sexual function. The patient’s functional status also showed steady improvement, as reflected by the spinal cord independence measure (SCIM). Our patient had a 45/100 score at admission and was discharged with a 66/100 score. Sixteen months after his infarct, the score was 74/100, with most significantly contributing to the functional disability being his bowel and bladder status. We found only one case to provide functional scale at least once, but not with SCIM [27].

The importance of the just mentioned issues is further stressed for pediatric FCE cases, which are being increasingly diagnosed in recent decades, with most of them surviving the acute infarct [9,11,28]. This translates into a young population of still maturing and developing individuals with special age-specific and long-term needs requiring special attention. Our patient successfully demonstrated consistent and high motivation for improving his motor status and associated functional goals. However, at the same time, low adherence to bladder management recommendations led to an opposite trend and functional deterioration.

### 3.5. Limitations

The assumption of FCE as the most likely underlying cause for our patient’s acute myelopathy was made eight days after the acute admission. This was enabled only after achieving a sufficient amount of clinical, laboratory, and radiological correlates to rule out the more commonly encountered pediatric etiologies for non-traumatic acute myelopathy while at the same time supporting FCE as being the most probable cause. It is important to note that, in our case, as is in all of the non-demised FCE suspected cases reported to date, this diagnosis was not achieved by the solely available GS measure of histopathological confirmation. As such, this is not a definite diagnosis, and the acute myelopathy might be otherwise well explained by a different etiology [7,11]. This is not unlikely, as previously reported apparently “idiopathic” spinal cord infarct cases in young individuals were eventually diagnosed with coagulation abnormalities due to rare genetic mutations [34]. Moreover, it is important to acknowledge that currently suggested clinical diagnostic criteria for FCE are mainly based upon partial and inconsistent data pooled from a scarce amount of case reports, which naturally were reported in a highly heterogeneous manner. This issue further undermines the level of certainty achieved by clinically diagnosing FCE on the grounds of the existing literature.

### 3.6. Conclusions

Our patient demonstrated a typical clinical course, which was characterized by rapidly progressive neurological deficits that occurred amidst physical activity. Acute MRI findings of T2WI signal hyperintensity without contrast enhancement at the anterior cord, Schmorl’s nodes, and minor horizontal disc herniations at several spinal column levels strongly support FCE. Long-term gains in neurological, motor, and functional outcomes could be explained by his initial injury classification of T4 AIS C, while also by his adherence to physical therapies and training. While it is possible for neurological deterioration to account for the deterioration in bladder function, low adherence to long-term management recommendations could also account for this.

### 3.7. Patient Perspective

“During my inpatient rehabilitation, I had physical therapy sessions that lasted about 40 min. The therapists included me in the treatment plan, though at times, some treatment sessions were confusing for me as I did not fully understand the reason or purpose behind them. Being more involved in my treatment plans and decisions was a very helpful experience for me. I feel it would have been even more helpful if I had been more involved. In the beginning, I was confused about functional and neurological outcomes, which made it hard to understand how they were affecting my recovery. Understanding these issues better helped me to better understand the purpose of various interventions and outcomes. During the inpatient stay, I met a young individual who experienced a similar event, and progressed and improved significantly. This meeting gave me hope, strength, and a positive build-up of expectations. However, as time progressed, I saw more limited improvement than expected. I realized that my improvement was more limited than I thought, which added some confusion and uncertainty as to what my final prognosis would be. I initially believed this was temporary, and I expected a full recovery. However, by now I have accepted my “handicap” and am more concentrated on improving what I still can”.

## Figures and Tables

**Table 1:** Long-term follow-up of patient’s neurological, functional, and urinary system status.

Diagnostic Studies Assessments	Time after Infarct			
	16 Days	2.5 Months	4 Months	16 Months
**ASIA exam**				
UERMS	25	25	25	25
UELMS	25	25	25	25
UEMS Total	50	50	50	50
LERMS	0	7	7	18
LELMS	18	25	25	25
LEMS Total	18	32	32	43
LTR	39	56	56	56
LTL	39	56	56	56
LT Total	78	112	112	112
PPR	24	28	33	41
PPL	25	30	43	45
PP Total	49	58	76	86
VAC	Yes	Yes	Yes	Yes
DAP	Yes	Yes	Yes	Yes
NLI	T4	T4	T4	T4
AIS	C	D	D	D
SCI classification	T4 AIS C	T4 AIS D	T4 AIS D	T4 AIS D
**SCIM**	45	N/A	66	74
**UDS**				
Compliance	▪ Good ▪ Maintains low pressure with volume increase during bladder filling.	N/A	▪ DSD ▪ DO ▪ Reduced ▪ 90 cmH_2_O at 240 mL and 110–140 cmH_2_O upon voluntary effort	▪ DSD ▪ DO ▪ Reduced ▪ 60–80 at 200 mL and 100 cmH_2_O upon voluntary attempts effort.
Voluntary bladder contraction	No	N/A	Yes	Yes
Voluntary voiding	No	N/A	Able to produce inconsistent stream with interrupted voiding.	Able to produce inconsistent stream with interrupted voiding.
VUR	No	N/A	No	No

**Abbreviations**: AIS, ASIA impairment scale; ASIA, American Spinal Cord Injury Association; DAP, deep anal pressure; DO, detrusor overactivity; DSD, detrusor sphincter dyssynergia; LELMS, lower extremity left motor score; LEMS, lower extremity motor score; LERMS, lower extremity right motor score; LT, light touch; LTL, light touch left; LTR, light touch right; MS, motor score; N/A, not availble; NLI, neurologic level of injury; PP, pinprick; PPL, pin prick left; PPR, pin prick right; SCI, spinal cord injury; UDS, urodynamic studies; UELMS, upper extremity left motor score; UEMS, upper extremity motor score; UERMS, upper extremity right motor score; VAC, voluntary anal contraction.

## Data Availability

Data is contained within the article and Supplementary Material.

## Supplementary Materials

The following supporting information can be downloaded at: https://www.mdpi.com/article/10.3390/neurolint15040080/s1, Supplementary Figure S1: the CARE guidelines.

## References

[1] Gupta M., Chhabra H.S. (2020). Nucleus Polposus Embolism Causing Anterior Spinal Artery Occlusion: A Rare but Possible Cause of Fibrocartilaginous Embolic Myelopathy. Int. J. Spine Surg..

[2] Ke W., Chen C., Li S., Wang B., Lu S., Yang C. (2022). Clinically suspected fibrocartilaginous embolism: A case report and literature review. Int. J. Neurosci..

[3] Al-Farsi S.A., Al-Abri H., Al-Ajmi E., Al-Asmi A. (2023). Spinal Cord Infarct Due to Fibrocartilaginous Embolism in an Adolescent Boy: A Case Report and Literature Review. Cureus.

[4] Naiman J.L., Donohue W.L., Richard J.S. (1961). Fatal nucleus pulposus embolism of spinal cord after trauma. Neurology.

[5] Fedaravičius A., Feinstein Y., Lazar I., Gidon M., Shelef I., Avraham E., Tamašauskas A., Melamed I. (2021). Successful management of spinal cord ischemia in a pediatric patient with fibrocartilaginous embolism: Illustrative case. J. Neurosurg. Case Lessons.

[6] McLean J.M., Palagallo G.L., Henderson J.P., Kimm J.A. (1995). Myelopathy associated with fibrocartilaginous emboli (FE): Review and two suspected cases. Surg. Neurol..

[7] AbdelRazek M.A., Mowla A., Farooq S., Silvestri N., Sawyer R., Wolfe G. (2016). Fibrocartilaginous embolism: A comprehensive review of an under-studied cause of spinal cord infarction and proposed diagnostic criteria. J. Spinal Cord Med..

[8] Moore B.J., Batterson A.M., Luetmer M.T., Reeves R.K. (2018). Fibrocartilaginous embolic myelopathy: Demographics, clinical presentation, and functional outcomes. Spinal Cord.

[9] Yamaguchi H., Nagase H., Nishiyama M., Tokumoto S., Toyoshima D., Akasaka Y., Maruyama A., Iijima K. (2019). Fibrocartilaginous Embolism of the Spinal Cord in Children: A Case Report and Review of Literature. Pediatr. Neurol..

[10] Nakstad I., Randjelovic I., Bergan H., Evensen K. (2020). Fibrocartilaginøs emboli som årsak til arteria spinalis anterior-syndrom?. Tidsskr. Nor. Legeforening.

[11] Ahluwalia R., Hayes L., Chandra T., Maugans T.A. (2020). Pediatric fibrocartilaginous embolism inducing paralysis. Child’s Nerv. Syst..

[12] Riley D.S., Barber M.S., Kienle G.S., Aronson J.K., von Schoen-Angerer T., Tugwell P., Kiene H., Helfand M., Altman D.G., Sox H. (2017). CARE guidelines for case reports: Explanation and elaboration document. J. Clin. Epidemiol..

[13] Weidauer S., Nichtweiß M., Hattingen E., Berkefeld J. (2015). Spinal cord ischemia: Aetiology, clinical syndromes and imaging features. Neuroradiology.

[14] Sandson T.A., Friedman J.H. (1989). Spinal cord infarction. Report of 8 cases and review of the literature. Medicine.

[15] Nedeltchev K., Loher T.J., Stepper F., Arnold M., Schroth G., Mattle H.P., Sturzenegger M., Kanellopoulos G.K., Kato H., Hsu C.Y. (2004). Long-Term Outcome of Acute Spinal Cord Ischemia Syndrome. Stroke.

[16] Mateen F.J., Monrad P.A., Hunderfund A.N.L., Robertson C.E., Sorenson E.J. (2011). Clinically suspected fibrocartilaginous embolism: Clinical characteristics, treatments, and outcomes. Eur. J. Neurol..

[17] Bansal S., Brown W., Dayal A., Carpenter J.L. (2014). Posterior Spinal Cord Infarction Due to Fibrocartilaginous Embolization in a 16-Year-Old Athlete. Pediatrics.

[18] Tosi L., Rigoli G., Beltramello A. (1996). Fibrocartilaginous embolism of the spinal cord: A clinical and pathogenetic reconsideration. J. Neurol. Neurosurg. Psychiatry.

[19] New P.W. (2019). A Narrative Review of Pediatric Nontraumatic Spinal Cord Dysfunction. Top. Spinal Cord Inj. Rehabil..

[20] Wang J.-Z., Yang M., Meng M., Li Z.-H. (2023). Clinical characteristics and treatment of spinal cord injury in children and adolescents. Chin. J. Traumatol..

[21] Alexander R.T., Cummings T.J. (2003). Pathologic Quiz Case: Acute-Onset Paraplegia in a 60-Year-Old Woman. Arch. Pathol. Lab. Med..

[22] Ciceri E.F., Opancina V., Pellegrino C., Scarabelli A., Botturi A.G., Bersano A., D’arrigo S., Erbetta A., Chiapparini L. (2023). Fibrocartilaginous embolism: A rare cause leading to spinal cord infarction?. Neurol. Sci..

[23] Eid R., Raj A., Farber D., Puri V., Bertolone S. (2016). Spinal Cord Infarction in Hemoglobin SC Disease as an Amusement Park Accident. Pediatrics.

[24] Han J.J., Massagli T.L., Jaffe K.M. (2004). Fibrocartilaginous embolism—An uncommon cause of spinal cord infarction: A case report and review of the literature^1^. Arch. Phys. Med. Rehabil..

[25] Davis G.A., Klug G.L. (2000). Acute-onset nontraumatic paraplegia in childhood: Fibrocartilaginous embolism or acute myelitis?. Child’s Nerv. Syst..

[26] Liskova Z., Lehotska V., Liska M., Mikula P. (2018). Fibrocartilaginous Embolization—A Rare Cause of Spinal Cord Infarction: Case Report. J. Radiol. Case Rep..

[27] Shah S., Bryant P. (2018). Fibrocartilaginous emboli in the pediatric population: The role of rehabilitation in facilitating functional recovery. J. Pediatr. Rehabil. Med..

[28] Verhey L.H., Banwell B.L., Dulac O., Lassonde M., Sarnat H.B. (2013). Chapter 104—Inflammatory, vascular, and infectious myelopathies in children. Handbook of Clinical Neurology.

[29] Manara R., Calderone M., Severino M.S., Citton V., Toldo I., Laverda A.M., Sartori S. (2010). Spinal Cord Infarction Due to Fibrocartilaginous Embolization: The Role of Diffusion Weighted Imaging and Short-Tau Inversion Recovery Sequences. J. Child Neurol..

[30] Elsayh K.I., Zahran A.M., El-Abaseri T.B., Mohamed A.O., El-Metwally T.H. (2014). Hypoxia Biomarkers, Oxidative Stress, and Circulating Microparticles in Pediatric Patients with Thalassemia in Upper Egypt. Clin. Appl. Thromb./Hemost..

[31] Olgar S., Kara A., Hicyilmaz H., Balta N., Canatan D. (2010). Evaluation of angiogenesis with vascular endothelial growth factor in patients with thalassemia major. Pediatr. Int..

[32] Shitrit D., Tamary H., Koren A., Levin C., Bargil-Shitrit A., Sulkes J., Kramer M.R. (2008). Correlation of vascular endothelial growth factor with the severity of thalassemia intermedia. Blood Coagul. Fibrinolysis.

[33] Kerzhner O., Berla E., Har-Even M., Ratmansky M., Goor-Aryeh I. (2023). Consistency of inconsistency in long-COVID-19 pain symptoms persistency: A systematic review and meta-analysis. Pain Pract..

[34] Khoueiry M., Moussa H., Sawaya R. (2021). Spinal cord infarction in a young adult: What is the culprit?. J. Spinal Cord Med..

[35] Rodrigues M., Beça G., Almeida A., Natário I., Vilabril F., Pereira M., Barreto J., Dias L., Gandarez F. (2021). Spinal cord infarction in children: Can gymnastics be a cause?. J. Pediatr. Rehabil. Med..

[36] Reisner A., Gary M.F., Chern J.J., Grattan-Smith J.D. (2013). Spinal cord infarction following minor trauma in children: Fibrocartilaginous embolism as a putative cause. J. Neurosurg. Pediatr..

[37] Draganich C., Wenzel L.R. (2021). Fibrocartilagenous embolism case series: Is it a zebra?. Spinal Cord Ser. Cases.

[38] Roberts T.T., Leonard G.R., Cepela D.J. (2017). Classifications In Brief: American Spinal Injury Association (ASIA) Impairment Scale. Clin. Orthop. Relat. Res..

[39] Nas K., Yazmalar L., Şah V., Aydin A., Öneş K. (2015). Rehabilitation of spinal cord injuries. World J. Orthop..

[40] Van Middendorp J.J., Goss B., Urquhart S., Atresh S., Williams R.P., Schuetz M. (2011). Diagnosis and Prognosis of Traumatic Spinal Cord Injury. Glob. Spine J..

