# Supplementary material for: Acute Presentation and Long-Term Rehabilitation Follow-Up of Ischemic Myelopathy Due to Clinically Suspected Fibrocartilaginous Embolism in an Adolescent Male: A Case Report and Review
Source: Neurol Int. 2023 Oct 19;15(4):1273–89. doi: 10.3390/neurolint15040080 (PMC10594434; doi:10.3390/neurolint15040080)
Supplement: Supplementary file 1 [file neurolint-15-00080-s001.zip › neurolint-2633468-supplementary.pdf]

CARE Checklist of information to include when writing a case report

Topic	Item	Checklist item description	Reported on Line
Title	1	The diagnosis or intervention of primary focus followed by the words "case report"	Heading
Key Words	2	2 to 5 key words that identify diagnoses or interventions in this case report, including "case report"	After abstract
Abstract (no references)	3a	Introduction: What is unique about this case and what does it add to the scientific literature?	✓ abstract
	3b	Main symptoms and/or important clinical findings	✓ abstract
	3c	The main diagnoses, therapeutic interventions, and outcomes	✓ abstract
	3d	Conclusion—What is the main "take-away" lesson(s) from this case?	✓ abstract
Introduction	4	One or two paragraphs summarizing why this case is unique (**may include references**)	1st and 2nd paragraph of intro
Patient Information	5a	De-identified patient specific information.	1st para in case presentation
	5b	Primary concerns and symptoms of the patient.	as above
	5c	Medical, family, and psycho-social history including relevant genetic information	1st para in case presentation last sentence
	5d	Relevant past interventions with outcomes	not relevent
Clinical Findings	6	Describe significant physical examination (PE) and important clinical findings.	Acute hospitalization 1st para
Timeline	7	Historical and current information from this episode of care organized as a timeline	Figure 1
Diagnostic Assessment	8a	Diagnostic testing (such as PE, laboratory testing, imaging, surveys).	During entire spectrum of followup
	8b	Diagnostic challenges (such as access to testing, financial, or cultural)	During entire spectrum of followup
	8c	Diagnosis (including other diagnoses considered)	During entire spectrum of followup
	8d	Prognosis (such as staging in oncology) where applicable	non aplicable
Therapeutic Intervention	9a	Types of therapeutic intervention (such as pharmacologic, surgical, preventive, self-care)	During entire spectrum of followup
	9b	Administration of therapeutic intervention (such as dosage, strength, duration)	During entire spectrum of followup
	9c	Changes in therapeutic intervention (with rationale)	During entire spectrum of followup
Follow-up and Outcomes	10a	Clinician and patient-assessed outcomes (if available)	non-aplicable
	10b	Important follow-up diagnostic and other test results	During long term followup
	10c	Intervention adherence and tolerability (How was this assessed?)	During long term of followup
	10d	Adverse and unanticipated events	During acute care
Discussion	11a	A scientific discussion of the strengths AND limitations associated with this case report	Limitation section
	11b	Discussion of the relevant medical literature **with references**.	Across discussion section
	11c	The scientific rationale for any conclusions (including assessment of possible causes)	conclusion section
	11d	The primary "take-away" lessons of this case report (without references) in a one paragraph conclusion	conclusion and abstract
Patient Perspective	12	The patient should share their perspective in one to two paragraphs on the treatment(s) they received	provided
Informed Consent	13	Did the patient give informed consent? Please provide if requested (provided)	Yes ☑ No ☐

Supplementary Figure S1. the CARE guidelines [12].
